# Immune Checkpoint Inhibitor in Hepatocellular Carcinoma: Response Rates, Adverse Events, and Predictors of Response

**DOI:** 10.3390/jcm14031034

**Published:** 2025-02-06

**Authors:** Shekhar Swaroop, Sagnik Biswas, Shubham Mehta, Arnav Aggarwal, Umang Arora, Samagra Agarwal, Amitkumar Chavan, Baibaswata Nayak, Shalimar

**Affiliations:** Department of Gastroenterology and Human Nutrition Unit, All India Institute of Medical Sciences, New Delhi 110029, India; s.swaroop123456@gmail.com (S.S.); dr.sagnikbiswas@gmail.com (S.B.); mehta.shubham1493@gmail.com (S.M.); arnav272@gmail.com (A.A.); umangarora@gmail.com (U.A.); samagra.agarwal@gmail.com (S.A.); dramitchavan921@gmail.com (A.C.); baibaswat@gmail.com (B.N.)

**Keywords:** HCC, immunotherapy, cirrhosis, liver, cancer

## Abstract

**Background/Objectives:** Hepatocellular carcinoma (HCC) is the most common primary hepatic malignancy. Barcelona Clinic Liver Cancer (BCLC) guidelines recommend antiangiogenic agents with immune checkpoint inhibitors as first-line therapy for advanced HCC. We present our experience of treating HCC patients with Atezolizumab–Bevacizumab, their response rates, adverse events, survival, and response and survival predictors. **Methods:** This retrospective analysis included HCC patients diagnosed at All India Institute of Medical Sciences, New Delhi, India between July 2021 and April 2024 and receiving at least one dose of Atezolizumab–Bevacizumab. The primary outcome was overall response rate (ORR), comprising complete response (CR) and partial response (PR), as per mRECIST criteria. Secondary outcomes were overall survival (OS), progression-free survival (PFS), and predictors of response and survival. **Results:** Sixty-three patients were analyzed {mean age: 56.0 + 12.7 years; 82.5% males}. Forty-three (68.2%) patients had BCLC stage C HCC. Thirty-five (55.5%) patients belonged to Child–Pugh class A and 28 (44.5%) belonged to Child–Pugh class B. At 1 year, OS was 39% and PFS was 27%. Among 43 patients with data for radiological response, ORR was 48.8% (CR—9.3% and PR—39.5%) and DCR was 62.7% with stable disease (SD) in 13.9% of patients. PD occurred in 37.2% of patients. AFP response predicted radiological response, while Child–Pugh class and BCLC stage predicted survival. Adverse events were reported in 49.2% of patients. **Conclusions:** Our study shows slightly lower survival than previous studies with Child–Pugh class being the most important determinant of survival. AFP response predicts radiological response and not survival.

## Simple Summary

Liver cancer has high incidence and mortality rates. Most liver cancers are still diagnosed at a very advanced stage. At present, the treatment options are limited, with immune check point inhibitors (ICIs) being recommended as first-line treatment for advanced HCC. This present study helps us understand the response rates and survival advantage provided by ICIs and the patients most likely to benefit from the ICIs. Also, the study helps us to know the various adverse events associated with the use of ICIs.

## 1. Introduction

Hepatocellular carcinoma (HCC) is the most common primary hepatic malignancy and the fourth most common cause of cancer-related deaths globally [1]. The prevalence of HCC in India, is 2.27 per 100,000, with a reported mortality rate of around 2.21 per 100,000 [2]. According to the GLOBOCAN data, liver cancer resulted in 36,953 deaths in India in 2022, ranking 8th among all cancers, and contributing to 4.0% of cancer-related deaths [3]. Despite advancements in diagnostic modalities and screening protocols, including radiological imaging, new biomarkers, next generation sequencing, artificial intelligence, molecular imaging, and liquid biopsy, a significant proportion of HCC cases are still diagnosed at advanced stages, with vascular invasion in 30% to 50% of cases and extra-hepatic metastasis in 20% to 40% of cases at time of presentation [2,4,5,6,7,8].

Treatment options for advanced HCC include antiangiogenic therapies (e.g., tyrosine kinase inhibitors [TKIs] and monoclonal antibodies) and immune checkpoint inhibitors [ICIs] (anti-programmed cell death protein-1 [anti-PD-1], anti-programmed death-ligand [PD-L1] and cytotoxic T-lymphocyte associated protein-4 [CTLA-4] inhibitors) [9]. The current Barcelona Clinic Liver Cancer (BCLC) prognosis and treatment strategy recommends systemic therapy combining antiangiogenic agents with immune checkpoint inhibitors such as Atezolizumab–Bevacizumab or Durvalumab–Tremelimumab as first-line therapy for patients with advanced HCC [10]. ICIs act by enhancing the T-cell-mediated immune response. Combining Atezolizumab (anti-PD-L1) with bevacizumab (anti-vascular endothelial growth factor [VEGF]) increases the efficacy of Atezolizumab by reversing VEGF-mediated immunosuppression and promoting T-cell infiltration into the tumor [11,12]. Despite the robust theoretical benefits of ICIs, the reported response rates and adverse events vary across studies ranging from 20 to 30% [13,14,15].

At present, there is uncertainty regarding the duration of therapy which depends on multiple factors like the clinical response, development of immune-related adverse events (iRAEs) and financial constraints [13,14]. There is also uncertainty regarding early predictors of clinical response and survival, as well as de-escalation once a clinical response is achieved [14,15,16,17]. Several biomarkers have been proposed as predictors of treatment response and survival [15,16,17]. High PD-L1 expression, tumor mutational burden, gut microbiota composition, and early alpha-fetoprotein (AFP) reduction have been reported to be associated with a better response [14,17,18,19,20,21]. Baseline liver function parameters (serum bilirubin, albumin), neutrophil to lymphocyte ratio (NLR), AFP reduction, Child–Pugh class, ALBI grade, and response to ICIs have been linked to survival outcomes [21,22,23,24]. In this study, we present our experience of treating HCC patients with Atezolizumab–Bevacizumab in terms of their response rate, adverse events, outcomes, and predictors of treatment response and survival.

## 2. Materials and Methods

### 2.1. Patients and Recruitment

In this retrospective analysis of a prospectively maintained database, all patients diagnosed with HCC at All India Institute of Medical Sciences, New Delhi, India between July 2021 and April 2024 were considered for inclusion. We excluded patients who were BCLC stage D and those who underwent liver resection or liver transplant. We further excluded patients who did not give consent for systemic therapies or who received only lenvatinib. Patients with uncontrolled hypertension or a history of autoimmune disorders were also excluded. The diagnosis of HCC was predominantly based on imaging {multiphase computerized tomography (CT) or magnetic resonance imaging (MRI)} with biopsy being performed in cases of diagnostic uncertainty. Ethical clearance was obtained from the institutional ethics committee (Ref. No.-IEC-768/12.11.2021, RP-78/2022).

### 2.2. Outcomes

The primary outcome was the overall response rate (ORR), comprising complete response (CR) and partial response (PR), defined as per modified response criteria in solid tumors (mRECIST) criteria [25]. The secondary outcomes were median overall survival (mOS), median progression-free survival (mPFS), etiology-wise response rate, predictors of response and survival, and adverse event rates (graded as per CTCAE v5).

### 2.3. Definitions

CR was defined as the disappearance of any intratumoral arterial enhancement in all target lesions. PR was defined as at least a 30% decrease in the sum of diameters of viable target lesions (defined by contrast enhancement in the arterial phase) relative to the baseline sum of the diameters. Progressive disease (PD) was defined as an increase of at least 20% in the sum of the diameters of viable target lesions, taking as reference the sum of the diameters of viable target lesions recorded since the treatment started. Stable disease (SD) was defined as any cases that did not qualify for either partial response or progressive disease [25]. Disease control rate (DCR) included the sum of CR, PR, and SD. Progression-free survival (PFS) was defined as the time period between the date of the first dose of ICIs to the date of death or the date of radiological evidence of tumor progression [26]. Overall survival (OS) was defined as the period between the date of the first dose of ICIs and the date of death. In patients with baseline AFP ≥ 20 ng/mL, AFP responders were defined as patients who reported >20% decrease in AFP values from baseline within 3 months of initiation of therapy with ICIs [27]. IrAEs were defined as adverse effects with a potential immunological basis including cutaneous, gastrointestinal, lung, endocrine, musculoskeletal, renal, nervous system, cardiovascular, and ocular toxicities [28].

### 2.4. Follow Up

All patients were followed up as outpatients in the Liver clinic at regular intervals as decided by the treating physician. All patients were treated with appropriate antiviral therapy for viral hepatitis. Diuretics and non-selective beta-blockers were prescribed, and other complications of cirrhosis were managed as per standard guidelines [29,30]. Radiological imaging was conducted and AFP levels were measured at 3-month intervals. Those who could not follow up in the clinic were followed up telephonically. ICI doses were stopped in cases of adverse events CTCAE grade 3 or higher, disease progression, decline in performance status [PS-3 or PS-4], tumor stage migration to BCLC-D or patient’s refusal to continue ICIs.

### 2.5. Statistical Analysis

The normality of the data was assessed using the Shapiro–Wilk test. Skewed continuous variables were expressed as median [interquartile range (IQR)], and non-skewed continuous data as mean with standard deviation (SD). Qualitative data were expressed as proportions (%). Normally distributed continuous variables were compared using Student’s *t*-test, a parametric test that assumes equal variances between groups. When the assumption of normality was not met, the Mann–Whitney U test, a non-parametric alternative, was applied to compare medians between two independent groups. Categorical variables were analyzed using the Chi-square test to evaluate the association between two or more groups. In cases where expected cell counts were small (less than 5 in any cell), Fisher’s exact test was used instead, as it is more reliable for small sample sizes. A *p*-value of <0.05 was considered to indicate statistical significance. Both mOS and mPFS were computed using Kaplan–Meier curves and compared with the Mantel–Cox log-rank test. Logistic regression analysis, including univariate and multivariate analysis, was performed to evaluate potential predictive factors for the response rates. Hazard ratios (HRs) for mortality were estimated using univariable and multivariable Cox regression analysis, including variables with *p* < 0.10 in the multivariable analysis. The data were analyzed using IBM SPSS Statistics software (version 20.0, Chicago, IL, USA), Medcalc software (version 15.11.4, MedCalc Software, Ostend, Belgium) and Stata software version 17.0, College Station, TX, USA (StataCorp).

## 3. Results

### 3.1. Patient Characteristics

**Figure 1:**
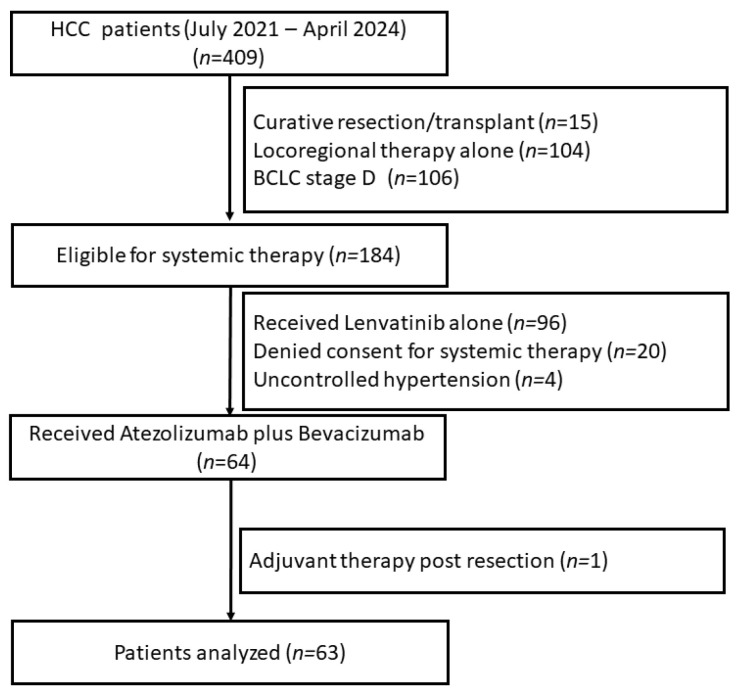
Flow chart of the study.

A total of 409 patients with a diagnosis of HCC attended the liver clinic at our center between July 2021 and April 2024. Three hundred and forty-five patients did not receive Atezolizumab and Bevacizumab for various reasons as given in Figure 1. Sixty-four patients received Atezolizumab–Bevacizumab. After excluding one patient who received Atezolizumab–Bevacizumab as adjuvant therapy post-resection, sixty-three patients were included for analysis (Figure 1). Mean age was 56.0 ± 12.7 years and 52 (82.5%) were males. The most common etiology of liver disease was viral hepatitis (*n* = 26, 41%) followed by metabolic dysfunction-associated steatotic liver disease (MASLD) (*n* = 15, 23.8%). Forty-three (68.2%) patients had BCLC-C HCC while nine (14.2%) had BCLC-A and eleven (17.4%) patients had BCLC-B HCC. All patients had cirrhosis, with Child–Pugh class A (55.5%) and B (44.4%). The median bilirubin and international normalized ratio (INR) levels were 0.97 (0.67–1.6) and 1.17 (1.09–1.27). Diabetes mellitus was the most common comorbidity and was present in 22 (34.9%) patients. The median number of doses of ICIs received was 4 (2–7). Baseline characteristics are shown in Table 1.

A total of 29 (46.0%) patients received ICIs alone, whereas 34 (54.0%) had received locoregional/TKI prior to starting ICIs in 31 (49.2%)/15 (23.8%) patients, respectively. The median model for end-stage liver disease (MELD) score was 9.3 (8.3–12.2) in those who received ICIs alone whereas the median MELD score was 8.4 (7.6–10.5) in those who received prior locoregional/TKI (Table 2).

**Table 1:** Baseline characteristics.

Characteristics	*n* = 63
Age (Mean, SD), years	56.0 (12.7)
Sex (Males, %)	52 (82.5%)
Etiology of cirrhosis	
Alcohol	08 (12.6%)
MASLD	15 (23.8%)
Viral hepatitis	26 (41.2%)
Others	14 (22.2%)
BCLC stage	
A	9 (14.2%)
B	11 (17.4%)
C	43 (68.2%)
Child–Pugh class	
A	35 (55.5%)
B	28 (44.4%)
Immunotherapy alone	29 (46.0%)
Prior therapy (Locoregional therapy/TKIs)	34 (54.0%)
Prior therapies	34 (54.0%)
Locoregional therapy *	31 (49.2%)
Oral TKI (Sorafenib/Lenvatinib)	15 (23.8%)
MELD	8.7 (8.1–11.1)
Hemoglobin (g/dL)	12.2 (10.7–13.4)
Total leucocyte count (/mm^3^)	5585 (3550–7400)
Platelets (×10^3^)	145 (89–197)
Urea (mg/dL)	24 (18.3–30)
Creatinine (mg/dL)	0.8 (0.6–0.9)
Bilirubin (mg/dL)	0.9 (0.6–1.6)
Alanine aminotransferase (U/L)	49 (32–89)
Aspartate aminotransferase (U/L)	52 (42–90)
Alkaline phosphatase (IU/L)	166 (124–222)
Albumin (g/dL)	3.8 (3.4–4.3)
International normalized ratio	1.2 (1.1–1.3)
ALBI grades	
1	27 (42.8%)
2	34 (53.9%)
3	2 (3.1%)
Diabetes mellitus	22 (34.9%)
Number of ICI doses, median (IQR)	4 (2–7)

* Locoreginal therapy includes Radiofrequency ablation, Microwave ablation, Transarterial chemoembolization, Transarterial radioembolization or stereotactic body radiation therapy (SBRT). Data presented as median (IQR)/*n* (%) unless mentioned otherwise. SD, standard deviation; MASLD, metabolic dysfunction-associated steatotic liver disease; BCLC, Barcelona Clinic Liver Cancer; TKIs, Tyrosine kinase inhibitors; MELD, model for end-stage liver disease; ALBI, albumin–bilirubin; ICI, immune checkpoint inhibitor, IQR; interquartile range.

**Table 2:** Disease severity and clinical outcomes.

	Whole Cohort (*n* = 63)	Only Immunotherapy (*n* = 29)	Prior Therapy * (*n* = 34)
Child–Pugh class			
A	35 (55.5%)	15 (51.7%)	20 (58.8%)
B	28 (44.4%)	14 (48.3%)	14 (41.1%)
MELD (Median, IQR)	8.7 (8.1–11.1)	9.3 (8.3–12.2)	8.4 (7.6–10.5)
Median (IQR) follow-up duration (days)	163 (90–309)	162 (90–264)	194 (105–310)
Median (IQR) overall survival (days)	316 (194–438)	195 (135–341)	413 (306–NE)
6-month overall survival (%, 95% CI)	66.9% (53.4–77.3%)	62.3% (40.8–77.9%)	70.6% (52.2–82.9%)
1-year overall survival (% 95% CI)	39.3% (23.5–54.8%)	27.9% (10–49.3%)	50.5% (27.2–69.8%)
Median (IQR) progression-free survival (days)	194 (124–309)	194 (105–NE)	240 (116–264)

Data presented as median (IQR)/*n* (%) unless mentioned otherwise. * Prior therapy includes locoregional therapy/TKIs received prior to starting of ICI. MELD, model for end-stage liver disease; NE, not estimable.

### 3.2. Characteristics of HCC

The median size of the HCC was 7.1 (4–10) cm with 26 (41.2%) patients having a single lesion and 16 (25.3%) patients having multifocal HCC (Table 3). Tumor in vein (TIV) was present in 32 (50.7%) patients, with 17 (26.9%) patients having main portal vein thrombosis and the rest having branch portal vein thrombosis. Twenty-one (33.3%) patients had metastatic HCC, with lungs (34.7%) and lymph nodes (34.7%) being the most common site of the tumor metastasis. Median AFP at presentation was 398 (40.3–10,000) ng/mL.

### 3.3. Clinical Outcomes

**Figure 2:**
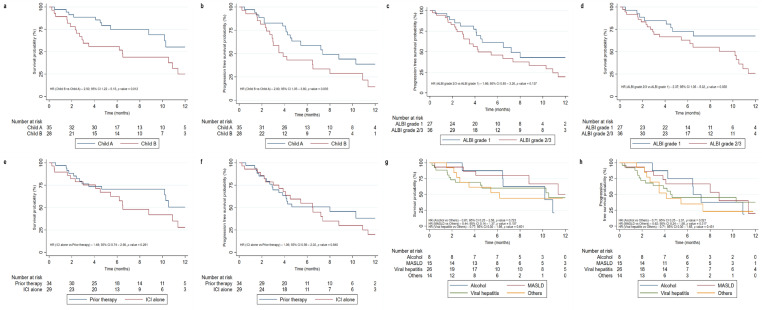
Kaplan–Meier curve showing a comparison of overall survival and progression-free survival among Child–Pugh classes (**a**,**b**), ALBI grades (**c**,**d**), those who received prior therapy vs. immunotherapy alone (**e**,**f**), and various etiologies of liver disease (**g**,**h**).

The median follow-up duration was 5.4 (3.0–10.3) months. Median OS was 10.5 (6.46–14.6) months and median PFS was 6.5 (4.1–10.3) months. The 6-month OS overall survival was 66.9%, while the 1-year OS overall survival was 39.3%. The PFS was 52.4% at 6 months and 26.8% at 1 year (Table 2). For 29 patients receiving only ICIs, no significant difference in OS was observed compared to those who received prior therapy (HR: 1.48; 95% CI, 0.74–2.98; *p* = 0.261) (Figure 2, Table S1). No difference was seen in OS and PFS across various etiologies. Similarly, no difference was seen in OS and PFS among those who developed irAE vs. those who did not develop irAE (Figure S1).

### 3.4. Radiological Response

Forty-three patients underwent repeat imaging for assessment of radiologic response; of the remaining twenty patients who did not undergo radiological reassessment, fourteen patients died before scheduled imaging, and six were lost to follow-up. Among the 43 patients who had undergone radiological response assessment, the proportion of patients with advanced liver disease (Child–Pugh B) was higher (Table S2). Out of the 43 patients, ORR was seen in 21 (48.8%) patients {CR: 4 (9.3%) and PR: 17 (39.5%)} and DCR was seen in 27 (62.7%) patients. SD was seen in six (13.9%) patients. PD was seen in 16 (37.2%) patients (Table 4). No significant difference was seen in response rates among various etiologies of liver disease (Table S3).

### 3.5. Clinical Outcomes Stratified by Residual Liver Function Assessment (Child–Pugh Class and ALBI Grades)

Median overall survival was 17.7 {8.8–Not estimable (NE)} months in Child–Pugh class A patients and 6.46 (2.4–11.3) months in Child–Pugh class B patients with a significant difference in OS between the two groups (Child–Pugh class B vs. A; HR, 2.55; 95% CI 1.2–5.1, *p* = 0.012). Median PFS was 7.4 (4.5–NE) months in Child–Pugh class A patients and 3.9 (2.7–8) months in Child–Pugh class B patients with a significant difference in PFS (Child–Pugh class B vs. A; HR, 2.00; 95% CI 1.0–3.8, *p* = 0.035) (Table S4, Figure 2). There was a significant difference in OS between patients having different ALBI grades (ALBI grade 2/3 vs. ALBI grade 1; HR, 2.37; 95% CI 1.1–5.3, *p* = 0.036). However, no significant difference was observed in PFS among the ALBI grades (ALBI grade 2/3 vs. ALBI grade 1; HR, 1.66; 95% CI 0.85–3.26, *p* = 0.137) (Table S5, Figure 2).

### 3.6. Outcomes in AFP Responders Versus Non-Responders

Among the 30 patients with available data on AFP response, 18/30 (60%) were classified as AFP responders, while 12/30 (40%) were classified as non-responders. No differences were seen between the two groups in terms of age, gender distribution, Child–Pugh score, or MELD score at presentation. During follow-up, seven patients (38.8%) died in the AFP responder group while four patients (33.3%) died in the AFP non-responder group, though this difference was not statistically significant (*p* = 0.757). Among patients with data available for both radiological and AFP response (25 patients), 15 were classified as AFP responders and 10 as AFP non-responders. A significantly greater number of patients among AFP responders had DCR as compared to non-responders (80% vs. 30%, *p* = 0.012) (Table S6).

### 3.7. Predictors of Overall Response

On univariate analysis, the only significant predictor of ORR was AFP response (Responders vs. non-responders; OR: 8.06; 95% CI, 1.53–42.31, *p* = 0.012). Tumor stage, size, portal vein involvement, Child–Pugh class, and ALBI score were not associated with ORR (Table S7).

### 3.8. Predictors of Overall Survival

On univariate analysis, significant predictors of mortality were BCLC stage, radiological response to ICIs, Child–Pugh class, and ALBI score. However, in a multivariable analysis, only radiological response to ICIs, BCLC stage, and Child–Pugh class at presentation remained significant predictors of mortality (Table S8).

### 3.9. Adverse Events

Adverse events of any grade, irrespective of causality, were reported by 31 (49.2%) patients, while grade 3 or higher adverse events occurred in 18 (28.5%) patients. The most common bleeding-related event was variceal bleeding in eight (12.6%) patients. Other adverse events included an increase in bilirubin in ten (16.0%) patients, aspartate aminotransferase (AST) rise > 3 times in seven (11.1%) patients and alanine aminotransferase (ALT) rise > 3 times in six (9.5%) patients, new onset or worsening of ascites in thirteen (20.6%) patients, and new onset HE in one (1.6%) patient. Adverse events are shown in Table 5. ICI treatment was stopped in 25 patients due to various reasons (fifteen—adverse events, five—progression of disease, one—refusal to continue ICIs, four—decline in performance status). Higher rates of adverse events were seen in Child–Pugh class B patients as compared to Child–Pugh class A patients; however, this difference was not statistically significant (60.7% vs. 40.0%, *p* = 0.102). Patients in ALBI grade 2/3 had significantly higher adverse events compared ALBI grade 1 (66.1% vs. 33.3%, *p* = 0.029) (Table S5). No difference in adverse events was observed between patients receiving immunotherapy alone and those receiving prior therapy (Table S1). The irAEs included skin rash, colitis, oral ulcers, and worsening liver functions, which occurred in 20 (31.7%) patients.

## 4. Discussion

In this retrospective study of 63 patients with BCLC stage A (14.2%), B (17.4%), and C (68.2%) patients we found a mOS of 10.5 months and mPFS of 6.5 months with standalone ICI therapy or combination therapy. The 6-month OS and PFS were 66.9% and 52.4%, respectively, while the 1-year OS and PFS were 39.3% and 26.8%, respectively. ORR, DCR, and PD were observed in 48.8%, 62.7%, and 37.2% of patients, respectively. AFP response correlated with the radiological response rate; however, a decline in AFP values >20% while on therapy did not result in improved survival. Response rates were similar across various etiologies of liver disease. Radiological response, BCLC stage, and Child–Pugh class were the only significant predictors of mortality in our cohort. Adverse events occurred in 44.4% of patients, with serious grade 3 or higher adverse events in 25.3% of patients.

A recent meta-analysis reported a pooled ORR of 33%, CR in 4%, and PR in 27% of patients [31]. The meta-analysis reported a pooled mOS and mPFS of 14.7 months and 6.6 months, respectively, similar to the IMbrave150 trial results [14]. While our ORR was consistent with these reports, the OS in our study was lower, likely due to inclusion of a higher proportion of Child–Pugh class B (45%) patients (as compared to the trials included in the meta-analysis which had predominantly Child–Pugh class A patients) and inadequate follow-up period. However, another meta-analysis which included 21 studies with 699 Child–Pugh class B patients treated with ICIs reported a notably lower mOS (5.49 months) and a reduced mPFS of 2.68 months [32], similar to our observations. The ORR and DCR in previous studies have been reported to be in the range of 25–45% and 65–85%, respectively, comparable to those observed in our study [33,34]. Additionally, our findings showed that AFP responders were significantly more likely to achieve a radiological response as compared to those who did not. Our findings corroborate earlier studies by Lee et al. and Teng et al. which reported that patients who receive ICIs and had an AFP response had a better radiological/clinical response and improved overall survival as compared to those who do not have an AFP response [24,35]. This supports the utility of AFP response as a surrogate marker for tumor response, particularly when frequent imaging assessments are not feasible. However, a limitation of this biomarker (AFP response) is that it cannot be evaluated before treatment and applies only to post-treatment data. However, we did not find an association between AFP response and survival outcomes, possibly due to the higher proportion of Child–Pugh class B patients and the limited data available for assessing AFP response.

Residual liver function plays an important role in determining the overall outcomes of HCC therapies. The available scoring systems for assessing residual liver functions include the MELD score, Child–Pugh class, and ALBI grade. Previous studies assessing the impact of Child–Pugh class on overall survival in Sorafenib-treated HCC patients have shown reduced survival with advanced Child–Pugh class [36,37,38]. Similarly, ALBI grade has been found to affect overall survival in patients treated with Sorafenib and Lenvatinib [39]. A large real-world study on Atezolizumab–Bevacizumab also reported reduced survival with higher Child–Pugh score [15]. Another study involving 67 patients found poor survival with advanced Child–Pugh class and ALBI grades, although response rates were similar between patients belonging to different Child–Pugh classes [33]. Adverse events were comparable between Child–Pugh class A and class B but were significantly higher in class C [33]. Our current findings corroborate these studies, showing similar radiological response rates across the Child–Pugh classes and ALBI grades. Survival was poorer in patients with higher Child–Pugh class and ALBI grades. Although adverse events were more frequent in Child–Pugh class B compared to Child–-Pugh class A, the difference was not statistically significant. ALBI grade 2/3 patients had a significantly higher rate of adverse events compared to ALBI grade 1 patients.

The response rates after ICI therapy across various etiologies of liver disease have shown inconsistent results in prior studies. The theoretical reason why response rates could be different across various etiologies of HCC may be attributed to differences in tumor immune microenvironment and intestinal microbiome [40]. A recent retrospective study demonstrated that ICIs accelerate the functional cure of hepatitis B in patients with hepatitis B-associated HCC [41]. The IMBrave 150 study and, subsequently, a meta-analysis including three studies (CheckMate-459, IMbrave150, and KEYNOTE-240) reported significantly improved OS in HCC of viral etiology as compared to non-viral etiology [42]. However, another meta-analysis reported a significant survival advantage in both viral and non-viral etiologies [43]. We did not find any significant difference in mortality and radiological response rates across patients of various etiologies in our cohort.

Previous studies have also reported a high incidence of adverse events associated with ICIs. In a meta-analysis involving 2831 patients, the incidence of any adverse events was 80% whereas the incidence of grade 3 and above adverse events was 35.4% [44]. In the long-term follow-up of the IMbrave150 study, adverse events of any grade occurred in 98% of patients whereas grade 3 and more adverse events occurred in 63% of patients [14]. Our current cohort revealed a much lesser rate of adverse events, maybe because of the small sample size and heterogeneity of definitions used.

Future research on ICIs in HCC should address gaps in knowledge, including their efficacy across varying liver dysfunction (Child–Pugh classes and ALBI grades) and etiologies (viral hepatitis, MASLD, and alcohol-related liver disease). Identifying biomarkers such as AFP, Protein induced by Vitamin K absence-II (PIVKA-II), NLR, and PD-L1 that can predict treatment response and survival. Studies should explore the relationship between irAEs and outcomes, and optimal ICI therapy duration, including strategies for treatment de-escalation after clinical response which is crucial for balancing efficacy, safety, and cost. Furthermore, the role of ICIs as neoadjuvant or adjuvant therapy in surgical or locoregional treatment settings should be studied to assess their impact on recurrence and survival. Additionally, combination therapies with locoregional treatments and tyrosine kinase inhibitors warrant investigation through large, multi-center studies.

Our study has several limitations. First, the retrospective nature of the study has several limitations including selection bias and misclassification bias. Second, the study included patients from a single center with a limited sample size, and future studies with a larger sample size are needed to validate our findings. There were missing data and some of the causes of deaths as well as adverse events may have been missed due to follow-up limitations. Another limitation of the study is short duration of follow-up. The use of prior locoregional/TKIs could have influenced the outcomes. Few patients could not continue the treatment for various reasons thus affecting the outcomes. Patients who were unable to undergo repeat radiological evaluation were likely those who experienced worsening clinical conditions after ICI therapy. This might have resulted in a selection bias, as only stable patients were included in the radiological response assessment.

## 5. Conclusions

In conclusion, our study shows poorer survival than the previous studies, with Child–Pugh class being the most important determinant of long-term survival. Future studies should focus on the efficacy of ICIs across liver disease severity and etiologies, and the identification of biomarkers for response and outcomes. Large multi-center well-designed studies are needed to explore the role of combination therapies.

## Figures and Tables

**Table 3:** Tumor characteristics.

Median (IQR) size, cm (largest observation)	7.1 (4–10)
Distribution of lesions among patients in our cohort (*n*, %)	
1	26 (41.2%)
2	14 (22.2%)
3	7 (11.1%)
>3	16 (25.3%)
Tumor in vein (TIV) *	32 (50.7%)
Main portal vein	17 (26.9%)
Branch portal vein	15 (23.8%)
Metastasis (No of patients) #	21 (33.3%)
Lungs	8 (34.7%)
Lymph nodes	8 (34.7%)
Adrenal	4 (17.4%)
Vertebral	2 (8.6%)
Omentum	1 (4.3%)
Alpha-fetoprotein (ng/mL)	398 (40.3–10,000)

Data presented as median (IQR)/*n* (%) unless mentioned otherwise. * Both MPV and branch PV thrombosis was taken as MPV thrombosis; # 1 patient had both lung and adrenal metastasis; 1 patient had both vertebral and lung metastasis; IQR, interquartile range.

**Table 4:** Radiological response.

Radiological response evaluated	43 (68.3%)
Overall response rate	21 (48.8%)
Complete response	4 (9.3%)
Partial response	17 (39.5%)
Stable disease	6 (13.9%)
Disease control rate	27 (62.7%)
Progressive disease	16 (37.2 %)
Could not be evaluated	20 (31.7%)
Died before scheduled MRI	14 (22.2%)
Did not come for follow-up MRI	6 (9.5%)

Data presented as *n* (%). Response assessment was conducted among 43 patients with at least one follow-up MRI.

**Table 5:** Adverse events.

Type of Adverse Event	*n* (%)	Severity (CTCAE v5)
Bleeding-related adverse events	12 (19.0%)	
Variceal GI bleed	8 (12.6%)	Grade 4/5
Non-variceal GI bleed	0 (0 %)	
IC bleed	2 (3.1%)	Grade 5
Epistaxis	2 (3.1%)	Grade 2
Oral ulcers	2 (3.1%)	Grade 2
Skin rashes	1 (1.6%)	Grade 2
Colitis	1 (1.6%)	Grade 2
Worsening Jaundice > 3x ULN	10 (16.0%)	Grade 3
AST increase		
>3x	7 (11.1%)	Grade 2
>5x	4 (6.3%)	Grade 3
ALT increase		
>3x	6 (9.5%)	Grade 2
>5x	1 (1.5%)	Grade 3
New onset/aggravation of ascites	13 (20.6%)	Grade 2
New onset/aggravation of HE	1 (1.6%)	Grade 3
Number of patients	31 (49.2%)	Any grade
Number of patients	18 (28.5%)	Grade 3 and above

Data presented as *n* (%). CTCAE, common terminology criteria for adverse events; IC, intracranial; ULN, upper limit normal; AST, aspartate transaminase; ALT, alanine transaminase; HE, hepatic encephalopathy.

## Data Availability

The datasets used and analyzed are during the current study and are available from the corresponding author on reasonable request.

## Supplementary Materials

The following supporting information can be downloaded at: https://www.mdpi.com/article/10.3390/jcm14031034/s1, Figure S1: Kaplan–Meier curve for (a) overall survival (OS) and (b) progression-free survival (PFS) comparing patient with immune-related adverse events (irAEs) vs. no immune-related adverse events (No irAEs). Table S1: Safety and efficacy as per prior therapy Table S2: Characteristics of patients in those who underwent repeat imaging vs. those who did not. Table S3: Etiology-wise radiological response rates (*n* = 63). Table S4: Baseline characteristics and efficacy of ICI across various Child-Pugh class. Table S5: Safety and effcacy of ICI as per the ALBI grade. Table S6: Outcomes as per the AFP response status. Table S7: Predictors of overall response rate (ORR). Table S8: Predictors of mortality.
