# Supplementary material for: Immune Checkpoint Inhibitor in Hepatocellular Carcinoma: Response Rates, Adverse Events, and Predictors of Response
Source: J Clin Med. 2025 Feb 6;14(3):1034. doi: 10.3390/jcm14031034 (PMC11818670; doi:10.3390/jcm14031034)
Supplement: Supplementary file 1 [file jcm-14-01034-s001.zip › Supplementary tables.pdf]

Supplementary table s1. Safety and efficacy as per prior therapy

	Prior therapy (*n*=34)	Immunotherapy alone (*n*=29)	*p*-value
6 months overall survival (95% CI)	70.6% (52.2% - 82.9%)	62.4% (40.8% - 78.0%)	0.574
1-year overall survival (95% CI)	50.5% (27.2%-69.8%)	27.9% (10% - 49.4%)	0.188
Adverse events	17 (50%)	14 (48.3%)	0.891
ALT rise >3x ULN	4 (11.7%)	2 (6.9%)	0.512
AST rise >3x ULN	3 (8.8%)	4 (13.7%)	0.532
Bilirubin rise > 3x ULN	6 (17.6%)	4 (13.7%)	0.677

CI, Confidence interval; ALT, Alanine transaminase; AST, Aspartate transaminase

Supplementary table s2. Characteristics of patients in those who underwent repeat imaging vs. those who did not.

	Evaluable (43)	Not evaluable (20)	*p*-value
Child Pugh class			0.025
A (*n*, %)	28 (65.1%)	7 (35.0%)	
B (*n*, %)	15 (34.8%)	13 (65.0%)	
MELD (median, IQR)	8.4 (8.1 - 10.2)	9.8 (8.2 - 12.5)	0.232

MELD, Model for end-stage liver disease

Supplementary table s3. Etiology-wise radiological response rates (*n*=63).

	Alcohol (*n*=8)	MASLD (*n*=15)	Viral hepatitis (*n*=26)	Others (*n*=22)	*p*-value
ORR	4 (50%)	7 (46.6%)	8 (30.7%)	2 (9.0%)	0.196
DCR	4 (50%)	9 (60.0%)	11 (42.3%)	3 (13.6%)	0.434
PD	2 (25%)	3 (20.0%)	6 (23.0%)	5 (22.7%)	0.818

Could not be evaluated	2 (25%)	3 (20.0%)	9 (34.6%)	6 (27.2%)	

Note: Data presented as n (%)

MASLD, Metabolic dysfunction associated steatotic liver disease; ORR, Overall response rate; DCR, Disease control rate; PD, Progressive disease

Supplementary table s4: Baseline characteristics and efficacy of ICI across various Child-Pugh class

	Child-Pugh class A (*n*=35)	Child-Pugh class B (*n*=28)	*p*-value
Age (Mean, SD), years	58.0 (11.8)	53.6 (13.4)	0.168
Sex (Males, %)	31 (88.5%)	21 (75.0%)	0.159
Ascites (*n*, %)	1 (2.8%)	22 (78.6%)	<0.001
Bilirubin (mg/dl)	0.8 (0.6 - 1.2)	1.4 (0.9 - 1.9)	0.006
Albumin (g/dl)	4.02 (3.5 - 4.4)	3.4 (3.1 - 3.9)	0.002
International normalized ratio	1.1 (1.1 - 1.2)	1.2 (1.1 - 1.4)	0.002
DCR (*n/N*, %)	9/28 (32.1%)	8/15 (53.3%)	0.348
Median (IQR) follow up duration (days)	163 (135 - 309)	173 (59 - 300)	0.220
6 months overall survival (95% CI)	74.7% (55.3% - 86.7%)	55.8% (35.5% - 72.0%)	0.062
1-year overall survival (95% CI)	55.2% (30.9% - 74.0%)	25.0% (8.8 - 45.3%)	0.020
Adverse events (*n*, %)	14 (40%)	17 (60.7%)	0.102

DCR, Disease control rate; IQR, Interquartile range; CI, Confidence interval

Supplementary table s5. Safety and effcacy of ICI as per the ALBI grade.

	ALBI grade 1	*ALBI grade 2/3	*p*-value
DCR (*n/N*, %)	14/21 (66.6%)	13/22 (59.0%)	0.607
Median (IQR) follow up duration (days)	195 (131 -295)	163 (83 - 310)	0.500
6 months overall survival (95% CI)	72.8% (51.2% - 86.2%)	62.9% (44.6% - 76.6%)	0.404
1-year overall survival (95% CI)	67.6% (45.1% - 82.4%)	25.8% (10.2% - 44.7%)	0.079
Adverse events (*n/N*, %)	9/27 (33.3%)	22/36 (61.1%)	0.029

DCR, Disease control rate; IQR, Interquartile range; CI, Confidence interval

*ALBI grade 2 and 3 were combined as ALBI grade 3 had only 2 patients

Supplementary table s6. Outcomes as per the AFP response status.

	AFP responders	AFP non-responders	*p*-value
Age (Mean, SD), years	56.1 (12.8)	51.4 (13.0)	0.338
Sex (Males, %)	17 (94.4%)	9 (75.0%)	0.125
MELD (Median, IQR)	8.6 (8.0 - 11.8)	8.4 (7.8 - 9.5)	0.497
Child-Pugh score (Median, IQR)	6 (5-8)	6 (5-7)	0.708
Child-Pugh class			0.880
A (*n*, %)	10 (55.6%)	7 (58.3%)	
B (*n*, %)	8 (44.4%)	5 (41.7%)	
Death (n=30)	7/18 (38.8%)	4/12 (33.3%)	0.757
DCR (n=25)	12/15 (80.0%)	3/10 (30.0%)	0.012

SD, Standard deviation; AFP, Alpha-fetoprotein; IQR, Interquartile range; MELD, Model for end-stage liver disease; DCR, Disease control rate

Supplementary table s7. Predictors of overall response rate (ORR)

	OR (95% CI)	*p*-value

Age	1.01 (0.98 - 1.06)	0.416
Sex (Female vs Male)	1.17 (0.30 - 4.57)	0.815
Etiology<br>(MASLD vs other)	2.12 (0.64 - 6.98)	0.214
BCLC stage<br>A<br>B<br>C	 1<br>0.46 (0.072 - 3.03)<br>0.60 (0.13 - 2.60)	 <br>0.427<br>0.498
Size of tumor	1.05 (0.93 - 1.19)	0.358
NLR<br>NLR category (>2.77 vs <2.77)	1.07 (0.90 - 1.28)<br>2.63 (0.69 - 9.94)	0.383<br>0.152
PVT	1.1 (0.38 - 3.13)	0.859
AFP	0.99 (0.99 - 1.00)	0.617
AFP category<br>AFP (responders vs non responders)	8.06 (1.53 - 42.31)	0.01
Child pugh class<br>(B Vs A)	0.50 (0.16 - 1.48)	0.213
ALBI score	1.04 (0.85 - 1.27)	0.681
ALBI grade<br>1<br>2/3	 Ref.<br>0.75 (0.23 - 2.5)	0.650
MELD score	1.07 (0.95 - 1.20)	0.229

OR, Odds ratio; MASLD, Metabolic dysfunction associated steatotic liver disease; BCLC, Barcelona Clinic Liver Cancer; NLR, Neutrophil to lymphocyte ratio; PVT, Portal vein thrombosis; AFP, Alpha fetoprotein; ALBI, Albumin-bilirubin; MELD, Model for end stage liver disease

Supplementary table s8. Predictors of mortality

	Univariate analysis	Multivariate analysis

	HR (95% CI)	*p*- value	HR (95% CI)	*p*- value
Age	1.00 (0.98 - 1.03)	0.714		
Sex (Female vs Male)	1.44 (0.53 - 3.87)	0.469		
Etiology<br>(MASLD vs other)	0.53 (0.21 - 1.32)	0.173		
BCLC stage<br>A<br>B<br>C	 Ref.<br>1.25 (0.17 - 9.06)<br>4.79 (1.13 - 20.30)	 <br>0.824<br>0.033	 Ref.<br>1.81 (0.23 - 14.47)<br>12.29 (2.14 - 70.56)	 <br>0.573<br>0.005
Size of tumor	1.00 (0.92 - 1.09)	0.935		
NLR<br>NLR category<br>(>2.77 vs <2.77)	0.96 (0.85 - 1.09)<br>1.03 (0.44 - 2.43)	0.573<br>0.935		
PVT	1.86 (0.90 - 3.86)	0.092	0.45 (0.17 - 1.18)	0.105
AFP	1.00 (0.99 - 1.00)	0.262		
Clinical response<br>(ORR vs others)	0.19 (0.07 - 0.52)	0.001	0.17 (0.05 – 0.50)	0.001
AFP category<br>AFP (responders vs non responders)	1.16 (0.53 - 2.56)	0.699		
Child-Pugh class<br>(B vs A)	2.5 (1.22 - 5.15)	0.012	2.71 (1.11 - 6.56)	0.027
ALBI score	2.17 (1.18 - 3.99)	0.013	1.59 (0.71 - 3.57)	0.256
ALBI grade<br>1<br>2/3	 Ref.<br>2.38 (1.06 - 5.33)	0.036	1.08 (0.43 - 2.76)	0.863
MELD score	1.07 (0.95 - 1.20)	0.229		

AST	0.99 (0.99 - 1.00)	0.743		

CI, Confidence interval, HR, Hazard ratio; MASLD, Metabolic dysfunction associated steatotic liver disease; BCLC, Barcelona Clinic Liver Cancer; NLR, Neutrophil to lymphocyte ratio; PVT, Portal vein thrombosis; AFP, Alpha fetoprotein; ALBI, Albumin-bilirubin; MELD, Model for end stage liver disease; AST, Aspartate transaminase

Supplementary figure s1: Kaplan-Meier curve for (a)overall survival (OS) and (b)progression-free survival (PFS) comparing patient with immune related adverse events (irAE) vs no immune related adverse events (No irAE)
