# Supplementary figures and images for: Immune Checkpoint Inhibitor in Hepatocellular Carcinoma: Response Rates, Adverse Events, and Predictors of Response
Source: J Clin Med. 2025 Feb 6;14(3):1034. doi: 10.3390/jcm14031034 (PMC11818670; doi:10.3390/jcm14031034)

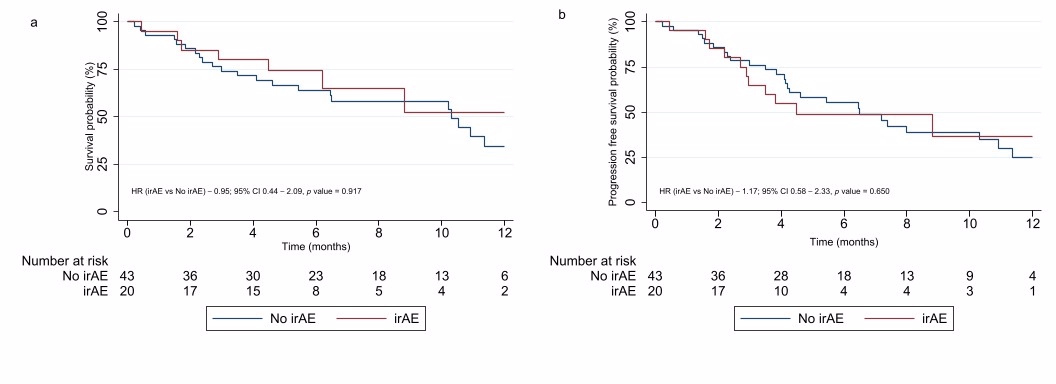

Supplement: Supplementary file 1 [file jcm-14-01034-s001.zip › Supplementary figure 1.jpg]
